# Three-dimensional optimization of squealer-tip for a transonic axial-flow compressor rotor blade

**DOI:** 10.1016/j.heliyon.2023.e23665

**Published:** 2023-12-21

**Authors:** Mojtaba Heidarian Shahri, Saeid Habibzadeh, Ali Madadi

**Affiliations:** Amirkabir University of Technology, Tehran, Iran

**Keywords:** Squealer-tip, Tip leakage, Performance map, Optimization, Axial compressor

## Abstract

This research represents an innovative method for geometry generation of the squealer-tip in axial compressor rotors and its exploit in a numerical optimization process to obtain a better stage performance. For this purpose, the NASA Rotor-67 transonic compressor rotor blade is used as a test case to study the aerodynamic performance using computational fluid dynamics. The validation was performed for the characteristic map at the design speed and the comparison with the experimental results indicates excellent matching and high adaptability of the numerical method. An ingenious method of producing squealer tip for an axial compressor rotary blade is presented in this article, which is capable for locally shaping both suction and pressure surface geometry at a desired spanwise location simultaneously, while keeping the tip clearance at its value of the baseline NASA Rotor-67 geometry. In this method, control points are used to produce the starting spanwise location of the squealer, and modify the depth of the squealer geometry. The L-27 orthogonal array of the Taguchi method as the Design of Experiment (DOE) has been used to investigate the sensitivity of the aerodynamic results in three performance points of the choke, design and near stall regions, in relation to the design variables of the squealer. The generated database in the sensitivity analysis was used to train artificial neural networks to replace the CFD solutions with overwhelming run time. By coupling the genetic algorithm to the aforementioned neural networks and by applying penalties to maintain the minimum performance of the Rotor-67, enhancement of total pressure ratio, adiabatic efficiency, mass flow rate and even the surge margin was achieved. The main effect of the squealer is to modify the shape of blade tip vortices, and by more dissipation of energy in blade tip area and reduced equivalent flow area in this region, finally results in improved overall mass flow rate, total pressure ratio, adiabatic efficiency and surge margin by 0.58 %, 0.36 %, 0.19 % and 4.81 % respectively, at design point.

## Nomenclature

a_1,_ a_2,_ a_3_: Coefficients of squealer depth spline for pressure side
a_4,_ a_5,_ a_6_: Coefficients of squealer depth spline for suction side
a_7_: Coefficients of squealer height
$\bar{A_{1}},\bar{A_{2}},\bar{A_{3}}$: The minimum, maximum and middle of Coefficient
ANN: Artificial neural network
C.F.: Correction factor
coeff: Coefficient of squealer-tip
Cp: Pressure coefficient
DOE: Design of experiments
k: The Ratio of Specific Heats
LE: Leading edge
$\dot{m}$: Mass Flow Rate [kg/s]
OA: Orthogonal array
Opt: Optimized
P: Pressure
PP: Participation percentage
PR: Total pressure ratio
S: Sum
SM: Surge margin
SOF: Square of factor
Sql: Squealer
T: Temperature [K]
TE: Trailing edge
TR: Temperature ratio
V: Velocity [m/s]
x, y, z: The x, y, z-coordinates
Y^+^: Y-plus

**Subscripts**
ad: Adiabatic
C: Chord
Ch: Choke
corr: Corrected
D: Design
in: Inlet
Leak: Leakage
NS: Near stall
out: Outlet
0: Stagnation property
PS: Pressure side
SS: Suction side
Tip: Tip section

**Greek symbols**
Δ: Difference
η: Adiabatic efficiency
V ×: Curl

## 1 introduction

The study of the effects of tip leakage flow in turbo-machines has undoubtfully been one of the major topics that have concerned the minds of great scientists of the aforementioned field.

In turbo-machines, tip leakage flow, caused by the pressure difference of suction and pressure surface of the blade, is unfavorable because of its adverse effect on pressure loss and blade exit wakes. The interaction of the tip leakage flow and the mainstream, or the shock wave in transonic compressors would worsen the case.

Cascade studies showed promising results when blade tip treatments were used. Winglets, which have been widely used in aircraft wings, have also proven to be beneficial in the compressor flow field and performance. Squealer tip is another passive control technique for turbine and compressor blades, aiming to alter the flow structure and extract better performance from the machine. Cavity-type squealer tips in turbines have been presented in the industry to reduce the tip leakage loss in axial turbines [1,2].

The squealer tip effect on the design and off-design performance of the compressor has been of great importance for researchers [3,4]. In 2016, Han Shaobing and Zhong Jinjun, numerically studied the effects of pressure and suction side winglets on the overall performance, flow structure, and stability of NASA rotor-37 blade. With a small penalty in efficiency, they reached a 33.74 stall range extension using a pressure side winglet, while the pressure side winglet was proven to be ineffective in their research. The alleviation of the interaction between tip leakage flow and shock waves was perceived to be the main reason for the mentioned performance improvement [5].

Squealer tips are indicated to be beneficial even in centrifugal compressors. In 2017, Riccardo Da Soghe et al. published their results of the study of the effects of squealer impellers on the performance of a centrifugal compressor. They found that the implementation of squealer tip impellers positively impacts performance. In high flow coefficients, the squealer tip was proven to be more effective than the part load conditions [6].

In 2022, a cavity-type squealer on transonic centrifugal compressor impellers and its effects on aerodynamic performance and stall margin were analyzed by Zamiri et al. They reported that a proper cavity depth could make a 0.32 % gain in efficiency at the design point and a 1.02 % improvement in stall margin [7].

In 2017, Shivaramaiah et al. numerically investigated the effects of four different winglet configurations added to the compressor blade tip. They tried two winglets on the pressure side of the blade, one fully covering the blade tip chord and the other only to 50% of the blade tip chord, and the same approach was conducted to the suction side. The results indicated that winglets can alter spanwise blade loading, besides increasing the stage total pressure ratio. In their research, the stall margin was found to decrease reportedly because of more blockage towards the trailing edge in the tip region [1].

Fei Zeng et al. developed a method to experimentally analyze squealer tip effects in turbines, considering the relative motion of the casing, which plays a vital role in the flow structure. They also considered how incidence angle variation affects performance. A method for using low-speed experimental results to study high-speed turbines was proposed and a cavity-type squealer-tipped blade was analyzed as a test case in the experimental setup [2].

High-pressure turbine blade tips are highly sensitive to small geometry variations. In 2021 J. Vieira and a group of researchers at Oxford University, aimed to study the aero-thermal effects of welding bead on a squealer-tipped turbine blade which is effective on engine performance. In their research, they surveyed flow structures and vortices in leakage flow when a squealer is present [8].

Q. Zhao et al., in 2021 employed a tip winglet to the NASA rotor-37 blade to numerically investigate its effects on flow stability. Four tip treatment configurations were used in this research. The results showed a decrement of leakage flow in the blade tip in the pressure side tip winglet configuration, but an increment in the suction side tip winglet configuration. With no penalty in efficiency, they experienced an 11 % increase in stall margin, while the suction side configuration resulted in a 17 % decrease in stall margin, with significant deterioration of flow characteristics and structure [9].

Oxford University researchers in 2021, implemented a multi-objective genetic algorithm to optimize squealer tip geometry for a turbine cascade to analyze cooling effects. Aerodynamic efficiency, film cooling effectiveness, and the variation of blade surface temperature were the main objectives to be optimized [10].

W. Xu and his colleagues have recently studied how a winglet in a compressor tip would affect the performance and flow structure of a transonic compressor. They studied the exploit of winglets in different tip clearances in their research project, besides studies of the effect of the winglet height. The weakening of the kinetic energy of tip leakage flow and suppression of leakage vortex, which in this case helps to reduce the total pressure loss, were the major results of the research. They experienced a 3.3% and a 13.4% decrease in loss and leakage flow in the optimal geometry. They could also show that, with increasing tip clearance, the suppression effect of winglets increases first and then gradually decreases [11].

In this research, a comprehensive and innovative method for the geometry generation of squealer-tip in both suction and pressure surfaces of an axial compressor rotor blade has been described. In this method, two splines are used to produce the squealer depth of the suction and pressure surfaces, which enables the designer to automatically produce various types of squealer curves using a few control points. The tip clearance value was kept constant to better investigate the role of the squealer in performance. A control point has also been used to enable changing the height of the squealer in the spanwise direction within predefined limits. To investigate the effect of the squealer in choke, design, and near the stall zones, the research was carried out using Taguchi's orthogonal array, and the feasible areas of aerodynamic improvement were identified and the results of the database were used to train artificial neural networks. Coupling of the genetic algorithm and artificial neural networks finally resulted in the optimal squealer geometry, based on various performance parameters. The obvious results of this research are the simultaneous squealer in both suction and pressure surfaces, which have improved the adiabatic efficiency of design and near stall points. The improvement of the surge margin is also the result that can be investigated after the optimization process.

## 2 three-dimensional simulation

In this section, the three-dimensional CFD simulation of the test case is described.

### 2.1 NASA Rotor-67 test case

**Fig. 1:**
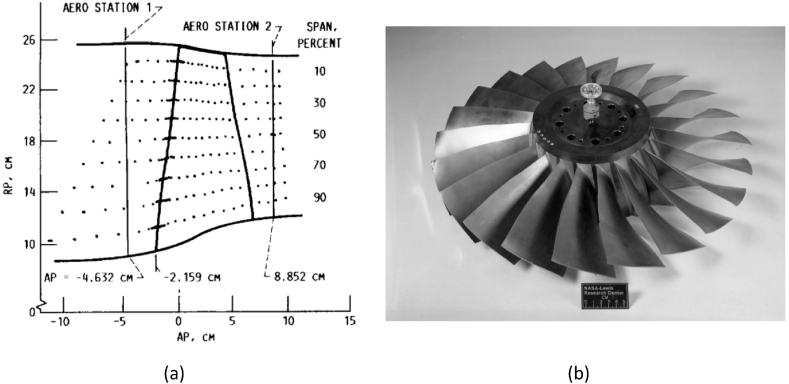
NASA Rotor-67: (a) Meridional view, (b) Three-dimensional view [12].

A numerical study was conducted on the geometry of NASA's Rotor-67 compressor, which provides a mass flow rate of 33.25 [kg/s] and a total pressure ratio of 1.63 at the design point, with a rotational speed of 16043 [rpm]. The three-dimensional and meridional views of this compressor are presented in Fig. 1. Stations 1 and 2, upstream and downstream of the rotor, are used to measure the rotor's overall performance.

The geometric and performance specifications of this compressor are summarized in Table 1, extracted from Ref. [12], which was used to validate the experimental test results.

**Table 1:** NASA Rotor-67 specification.

**Geometry specification**	Number of blades	22
	Inlet tip diameter [cm]	51.4
	Exit tip diameter [cm]	48.5
	Inlet hub-to-tip radius ratio	0.375
	Exit hub-to-tip radius ratio	0.478
	Hub solidity	3.11
	Tip solidity	1.29
	Rotor aspect ratio	1.56
**Performance values**	Rotational speed [rpm]	16043
	Total pressure ratio	1.632
	Mass flow rate [kg/s]	33.794
	Adiabatic efficiency	0.919
	Inlet tip relative Mach number	1.38
	Tip speed [m/s]	429

**Fig. 2:**
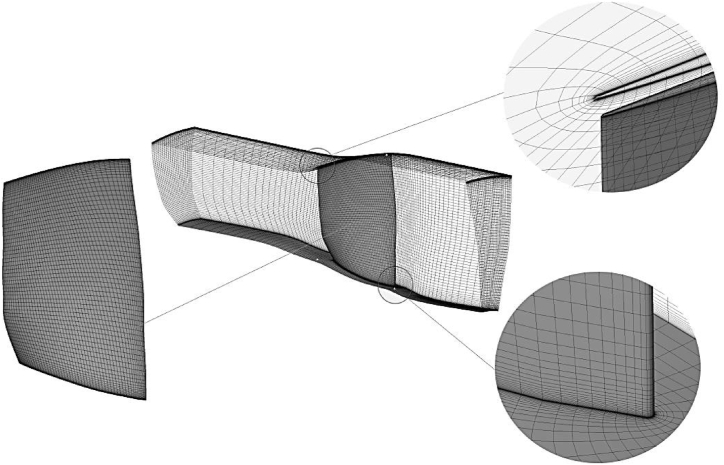
Structured grids for NASA Rotor-67.

**Fig. 3:**
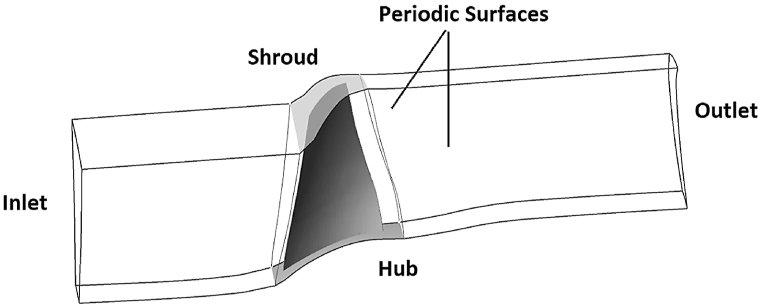
The boundary conditions for aerodynamic domain.

A three-dimensional aerodynamic analysis of the compressor blade was performed using the Reynolds-Averaged Navier-Stokes code (RANS) with structured grids comprising H and O blocks, as shown in Fig. 2. Side surfaces of the domain were set as periodic faces. In addition, the atmospheric conditions were used at the compressor inlet for the air ideal gas, and the static pressure condition was used at the outlet as presented in Fig. 3. The rotor domain is set to be rotary while the hub and shroud walls were set with a no-slip condition. The SST model was used as the turbulence model in the analysis.

**Fig. 4:**
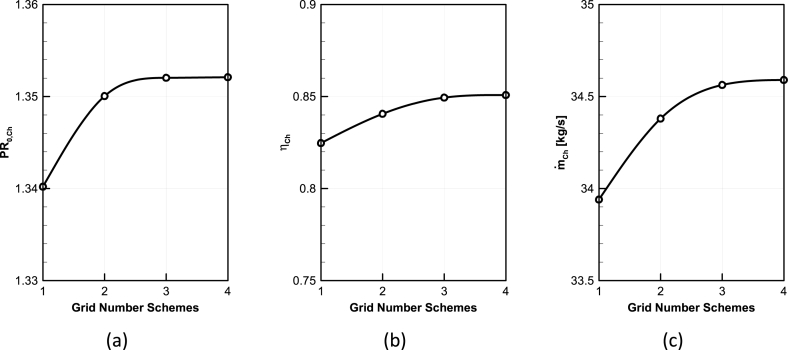
Grid study of CFD results, (a) Total pressure ratio, (b) Adiabatic efficiency, (c) Mass flow rate.

Four structured grids with 480k, 720k, 1360, and 1780k elements are generated for grid study. The comparison of total pressure ratio, mass flow rate and adiabatic efficiency for these four grids at choked point are demonstrated in Fig. 4.

**Fig. 5:**
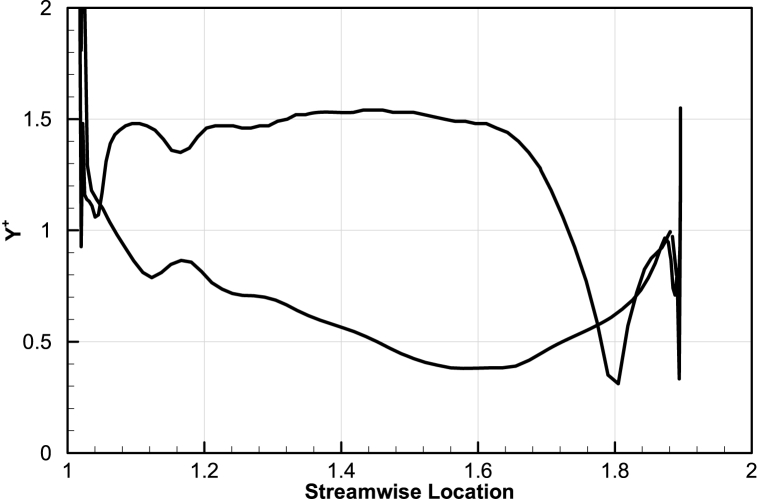
NASA Rotor-67 Y^+^ distribution.

Considering the results of the grid study, the grid no.3 can be chosen as the efficient case to continue the study. Fig. 5 depicts the distribution of blade Yplus in grid No.3 which in case verifies the validity of the turbulence model. The average distribution of the Y-plus value in the middle section of Rotor Blade grid No.3 is approximately 1, which is within the ideal range for the turbulence model used.

### 2.2 CFD solver validation

**Fig. 6:**
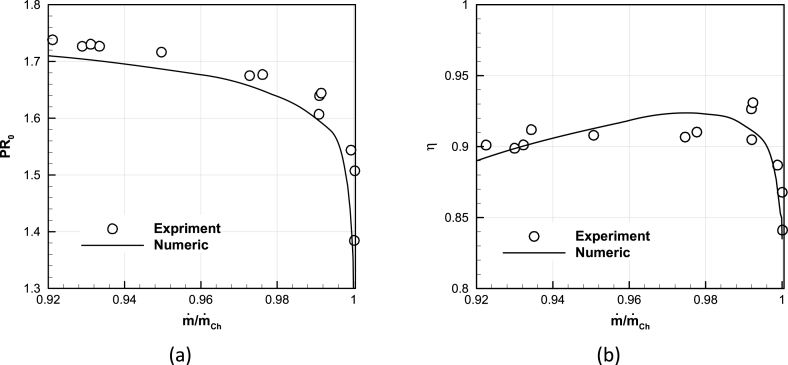
Validation of numerical and experimental results at design speed [12], (a) Total pressure ratio vs. non-dimensional mass flow rate, (b) Adiabatic efficiency vs. non-dimensional mass flow rate.

To validate the CFD solver, the performance maps from the numerical simulation at the design speed are compared to the experimental data and shown in Fig. 6 [12].

The minimum total pressure ratio obtained in the numerical method equals to 1.28 at the choke condition, with the maximum mass flow rate of 34.55 [kg/s] and the adiabatic efficiency of 84 %. The numerical simulations also resulted in a maximum Total pressure ratio of 1.67 near the stall region, with a minimum mass flow rate of 33.14 [kg/s] and an adiabatic efficiency of 88 %. The maximum adiabatic efficiency of the compressor is achieved at its design point and is 92.26 % with a total pressure ratio of 1.635 and a mass flow rate of 33.91 [kg/s].

**Fig. 7:**
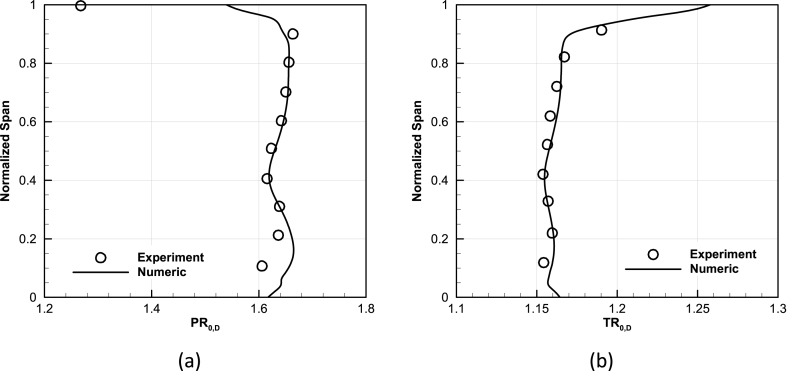
Validation of radial profile of performance parameters at design point, (a) Spanwise distribution of total pressure ratio, (b) Spanwise distribution of total temperature ratio.

Another validation perspective for the numerical results compared the available experimental data of total pressure ratio and total temperature ratio distribution from the hub to the shroud at the design point with the numerical results, as shown in Fig. 7. Capturing the complete trend of characteristic curves and radial profiles all proved the valid results of the numerical method.

## 3 squealer-tip geometry

In this research, an innovative method is used to produce the squealer tip geometry for the rotor blade which is applied for both suction and pressure surfaces. The effect of the squealer is investigated at choke, design and near stall conditions.

### 3.1 Parameterization

**Fig. 8:**
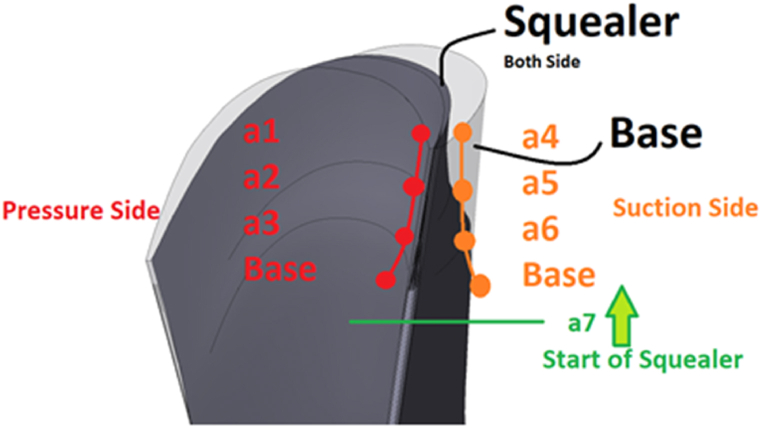
Schematic view of the squealer tip parameters in axial compressor rotary blade tip.

In this research, two spline curves are employed to generate squealer-tip geometry in the axial compressor blade, simultaneously in suction and pressure surfaces. Three control points define the depth of the squealer starting from an initial predefined span to the tip-span on the pressure side (a_1_, a_2_, and a_3_) and suction side (a_4_, a_5_, and a_6_). The aforementioned starting span is also an optimization parameter (a_7_), determining the starting location of the squealer in the spanwise direction. Fig. 8 demonstrates a schematic view of the squealer geometry in the rotary blade tip of an axial compressor.

A normalized coefficient is used to produce a thinner airfoil in the blade tip region. In this way, first the distance (${\Delta x,}_{PS-SS},{\Delta y,}_{PS-SS},{\Delta z,}_{PS-SS}$) of each point on the suction and pressure surfaces from the camber line is calculated (Eqn. (1)), then the related coefficient (Coeff) is multiplied to this distance; and at last the pressure/suction sides of new airfoil coordinates are calculated by addition or subtraction with camber ($x_{C},y_{C},z_{C}$) coordinates (Eqn. (2)

(1) $${\Delta x}_{PS-SS}=\frac{x_{PS}+x_{SS}}{2}\;{\Delta y}_{PS-SS}=\frac{y_{PS}+y_{SS}}{2}\;{\Delta z}_{PS-SS}=\frac{z_{PS}+z_{SS}}{2}$$

(2) $$x_{PS/SS}=x_{C}\;\underline{+}\;\frac{{\Delta x}_{PS-SS}}{2}\times Coeff\;y_{PS/SS}=y_{C}\;\underline{+}\;\frac{y_{PS-SS}}{2}\times Coeff\;z_{PS/SS}=z_{C}\;\underline{+}\;\frac{{\Delta z}_{PS-SS}}{2}\times Coeff$$

Finally, the three-dimensional geometry is generated by stacking the thinner airfoils on the tip of the blade.

Research on the optimal squealer was carried out in two separate simulations, for two-sided (suction-pressure surfaces) squealer and one-sided squealer.

**Fig. 9:**
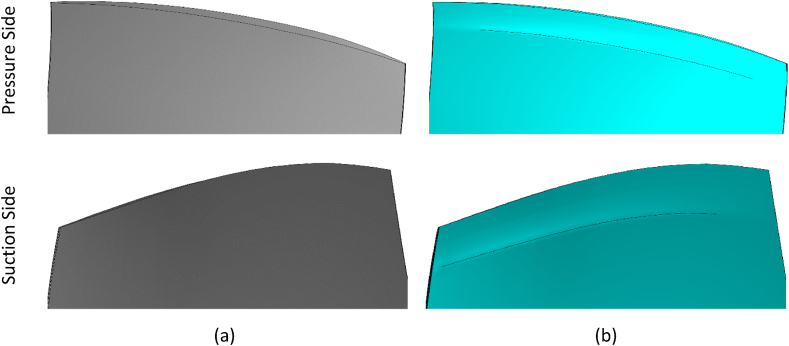
Comparison of geometries, (a) NASA Rotor-67, (b) Squealer-PS&SS configuration.

**Fig. 10:**
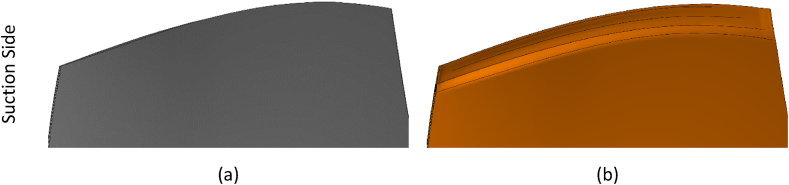
Comparison of geometries, (a) NASA Rotor-67, (b) Squealer-PS configuration.

**Fig. 11:**
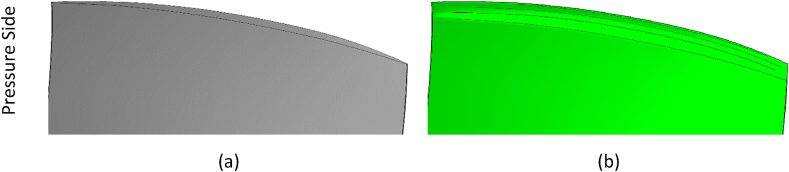
Comparison of geometries, (a) NASA Rotor-67, (b) Squealer-SS configuration.

Fig. 9 shows the squealer production for two-sided suction-pressure, while Fig. 10, Fig. 11 show the one-sided squealer production for a suction and pressure surface, respectively.

### 3.2 constraints

**Fig. 12:**
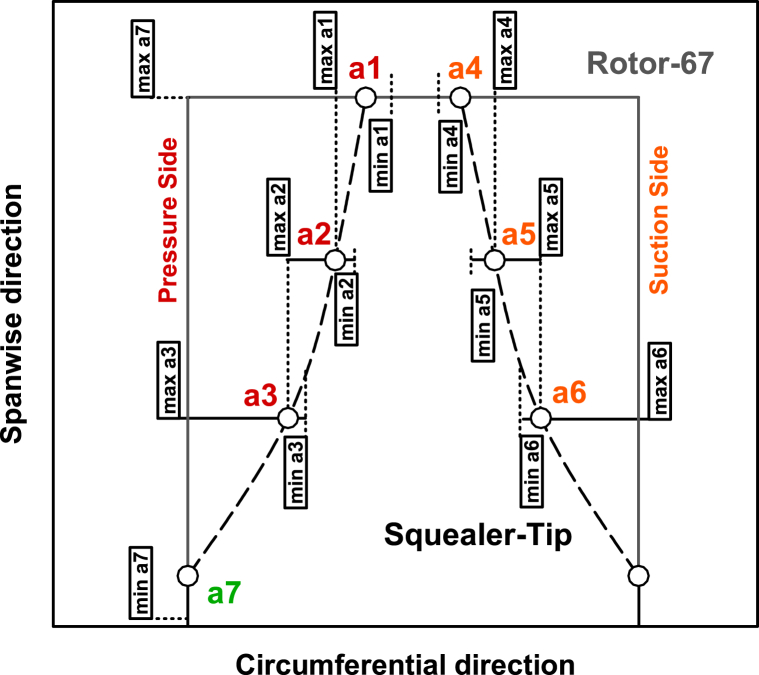
Schematic view of minimum and maximum limits of the squealer-tip coefficients.

For the design parameters, the constraints are considered to produce the geometries for the squealer tip rotor. These parameters are graphically presented in Fig. 12. Parameters a_3_ and a_6_ define the relative location of the first control point of the squealer geometry. For the parameters a_3_ and a_6_, the maximum value is set to be 1 which corresponds to the original airfoil without squealer. The parameters a_2_ and a_5_ are the relative distance of the second control points from the camber line to the first control point. A maximum value of 1 for these parameters means that the first and second control points are coincident. Similarly, parameters a_1_ and a_4_ define the relative distance of the third control points from the camber line to the second control points. Here, a maximum value of 1 means the coincidence of the second and the third control points.

The variable a_7_ also defines the spanwise location of the first profile for the squealer geometry. Its minimum and maximum values are set to be 90 % and 100 %, respectively. Table 2 shows the minimum and maximum limits of the squealer-tip coefficients. The coefficients are presented the relative percentage of airfoil thickness at each section.

**Table 2:** Minimum and maximum values of blade squealer-tip coefficients.

		Squealer-SS&PS		Squealer-SS		Squealer-PS	
Location	Coefficients	Minimum	Maximum	Minimum	Maximum	Minimum	Maximum
**Pressure side**	**a**_**1**_	0.70	1.00	0.10	1.00	1.00	1.00
	**a**_**2**_	0.60	1.00	0.10	1.00	1.00	1.00
	**a**_**3**_	0.50	1.00	0.10	1.00	1.00	1.00
**Suction side**	**a**_**4**_	0.70	1.00	1.00	1.00	0.10	0.10
	**a**_**5**_	0.60	1.00	1.00	1.00	0.10	0.10
	**a**_**6**_	0.50	1.00	1.00	1.00	0.10	0.10
**Span**	**a**_**7**_	0.90	1.00	0.90	1.00	0.90	1.00

Table 2 summarizes the minimum and maximum values for the defined design parameters.

### 3.3 objective functions

The objective functions of this research include three main performance parameters of a compressor including corrected mass flow rate, total pressure ratio and adiabatic efficiency according to equations (3), (4), (5).

(3) $${\dot{m}}_{corr}=\dot{m}\frac{\sqrt{T_{0\;in}/288.15}}{\sqrt{P_{0\;in}/101325}}$$

(4) $$\eta_{ad}=\frac{{\left(P_{0\;out}/P_{0\;in}\right)}^{\left(k-1\right)/k}-1}{T_{0\;out}/T_{0\;in}-1}$$

(5) $${\mathrm{PR}}_{0}=P_{0\;out}/P_{0\;in}$$

## 4 design of experiments

Design of experiments (DoE) refers to methods used to investigate and explore the effects of different design variables under various conditions on the results [13]. So, the design of experiment using Taguchi's approach has been used to study the sensitivity of different compressor performance parameters to variation of defined geometrical parameters mentioned in previous sections.

### 4.1 Taguchi DOE approach

Considering the number of design variables which are 3 parameters for pressure side, 3 parameters for suction side and 1 parameter for the starting location of squealer-tip, the L-27 OA, meaning 27 three-level factors on 7 design variables has been used to investigate the results. Table 3 demonstrates the L-27 OA [13].

**Table 3:** Taguchi L-27 orthogonal array.

Factor	1	2	3	4	5	6	7	8	9	10	11	12	13	14	15	16	17	18	19	20	21	22	23	24	25	26	27
**A**	1	1	1	1	1	1	1	1	1	2	2	2	2	2	2	2	2	2	3	3	3	3	3	3	3	3	3
**B**	1	1	1	2	2	2	3	3	3	1	1	1	2	2	2	3	3	3	1	1	1	2	2	2	3	3	3
**C**	1	1	1	2	2	2	3	3	3	2	2	2	3	3	3	1	1	1	3	3	3	1	1	1	2	2	2
**D**	1	1	1	2	2	2	3	3	3	3	3	3	1	1	1	2	2	2	2	2	2	3	3	3	1	1	1
**E**	1	2	3	1	2	3	1	2	3	1	2	3	1	2	3	1	2	3	1	2	3	1	2	3	1	2	3
**F**	1	2	3	1	2	3	1	2	3	2	3	1	2	3	1	2	3	1	3	1	2	3	1	2	3	1	2
**G**	1	2	3	1	2	3	1	2	3	3	1	2	3	1	2	3	1	2	2	3	1	2	3	1	2	3	1

Each row of the orthogonal array table corresponds to a trial. The numbers 1, 2 and 3 correspond to the minimum, medium and maximum values, respectively.

### 4.2 sensitivity analysis

The squealer-tip rotor geometry and grid generation and numerical simulation were performed for all the trials of DOE matrix, and the results of objective functions were extracted for the choke, design and near stall operating regions of design speed.

These performance parameters and their variables are utilized to perform the sensitivity analysis. In this way, the results are specified as y_1_ to y_27_ for which the subscript corresponds to the trial number. For each of the design variables, the average function is calculated using equation (6) in which $\bar{A_{1}}$ , $\bar{A_{2}}$ and $\bar{A_{3}}$ are the mean of the objective functions for minimum, maximum and medium case of variable A, respectively.

(6) $$\bar{A_{1}}={\frac{\sum y_{i}}{n}|}_{A_{1}},\bar{A_{2}}={\frac{\sum y_{i}}{n}|}_{A_{2}},\bar{A_{3}}={\frac{\sum y_{i}}{n}|}_{A_{3}}$$

(7) $$C.F.=\frac{{Sum}^{2}}{n_{total}}$$

(8) $${SoF}_{A}=\frac{A_{1}^{2}}{n_{A1}}+\frac{A_{2}^{2}}{n_{A2}}+\frac{A_{3}^{2}}{n_{A3}}-C.F\text{.}$$

(9) $${PP}_{A}=\frac{S_{A}}{\sum S}\times 100$$

The sum of all simulations is represented by the variable Sum, while n_total_ is the total number of experiments. The correction factor is determined with C.F. (Eqn. (7)). The sum of the squares of the factors related to each parameter is denoted by the SOF (Eqn. (8)). The participation percentage of each parameter is also determined by the PP (which is presented in Eqn. (9)) [13].

**Fig. 13:**
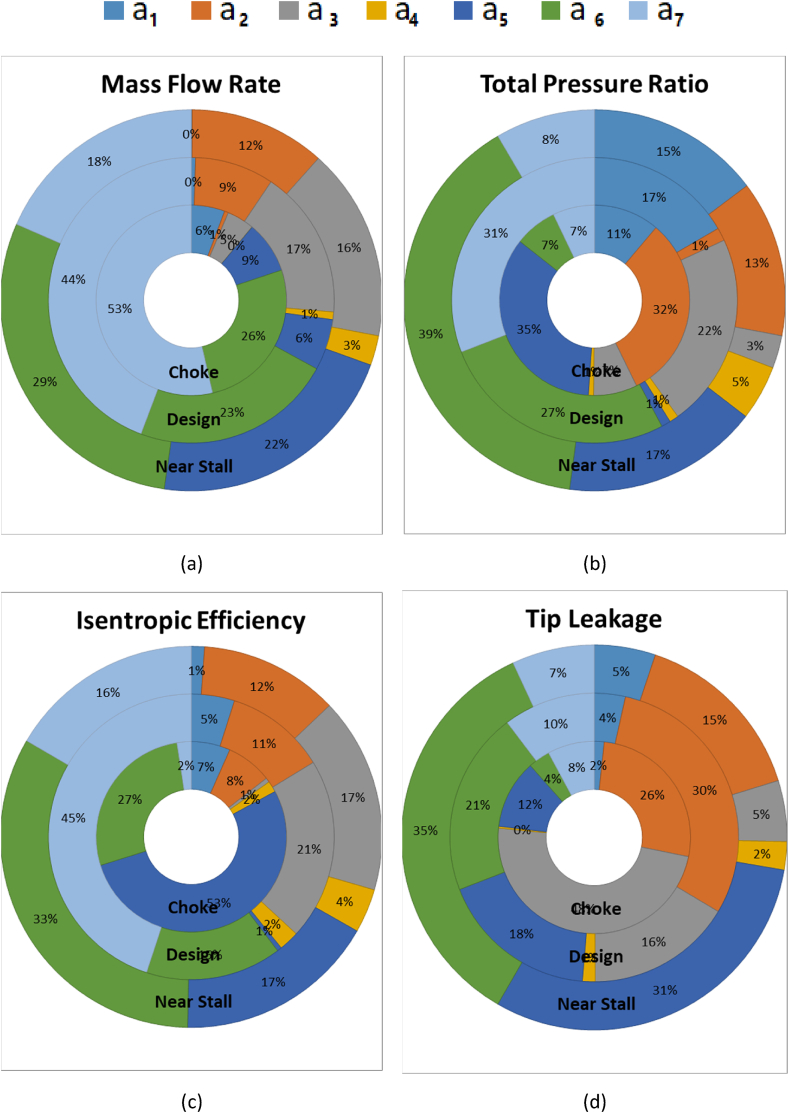
Sensitivity analysis of performance parameters: (a) Mass flow rate, (b) Total pressure ratio, (c) Adiabatic efficiency, (d) Tip leakage mass flow rate.

Having calculated these parameters, the effects of the design variables (three levels factor) on the objective functions have been obtained. Fig. 13 demonstrates the percentage of sensitivities of the objective functions.

The results of sensitivity analysis are summarized below.

• The control points of the suction surface and the start of the squealer (a_7_) have the greatest effect on the mass flow rate changes of all three operating points.
• In both suction and pressure surfaces, the starting point of the squealer tip primarily affects the on-design compressor total pressure ratio.
• The control point of the squealer height (a_7_) as well as the control points of the suction surface have a great effect on the adiabatic efficiency (design and near stall points) of the compressor.
• a_1_ and a_2_, mainly affect the two parameters of the leakage mass flow rate and the tip flow velocity.
• Parameter a6 appears to significantly affect all performance parameters in the near stall operating point.

## 5 optimization process

The optimization process includes the use of artificial neural networks for accurate prediction of the compressor performance and reduction of the CFD run time, coupling with the optimizer algorithm until reaching the desired objectives, and finally presenting the optimal results obtained, which has been explained in this section.

### 5.1 Methodology

Using Artificial Neural Network (ANN) as a powerful tool is a common method to use for optimization problems. The most important advantage of this optimization coupling is the reduction of the computational costs in the optimization algorithm cycle. The coupling of ANNs with the genetic algorithm has been widely used in the field of turbo-machinery optimization. Some examples include the research of Benini and Heidarian both for an axial compressor [14,15] and Ekradi et al. for a centrifugal compressor [16]. Here, the coupling of genetic algorithm and the artificial neural network is utilized to obtain optimal values of squealer-tip geometry parameters, which were defined in previous sections.

The results of the L-27 OA of Taguchi (used in the sensitivity analysis step) were used to train the neural network to be used to predict the values of mass flow rate, Total pressure ratio, and adiabatic efficiency for the three operating points of choke, design and near stall.

**Fig. 14:**
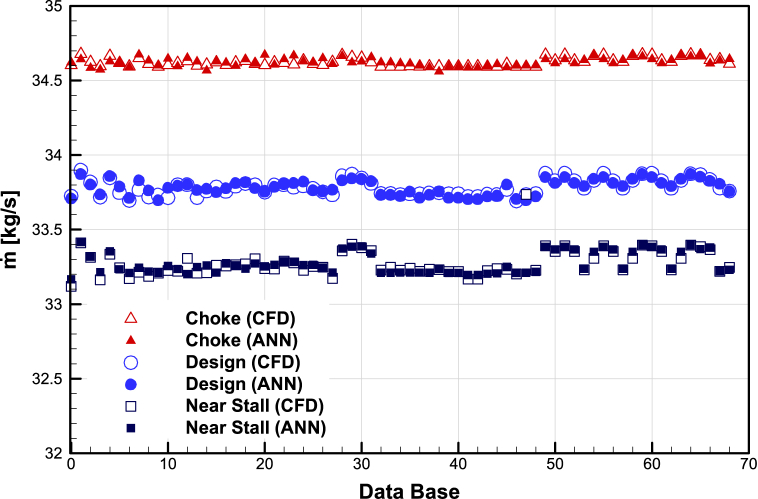
The prediction accuracy of mass flow rate of ANN with CFD.

**Fig. 15:**
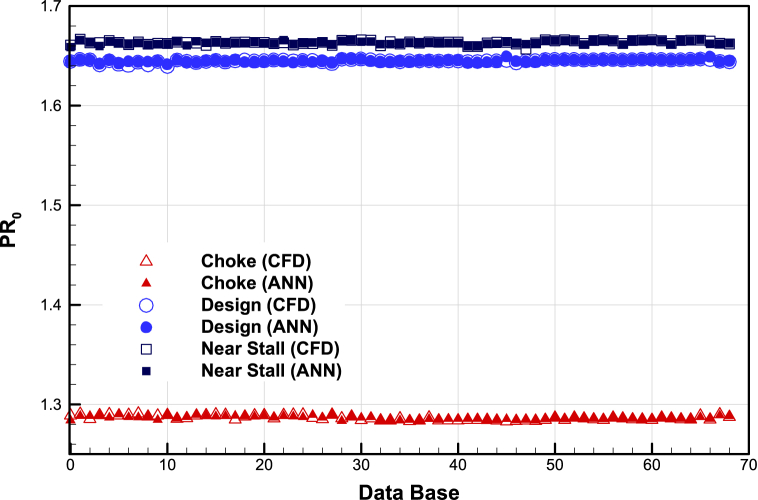
The prediction accuracy total pressure ratio of ANN with CFD.

**Fig. 16:**
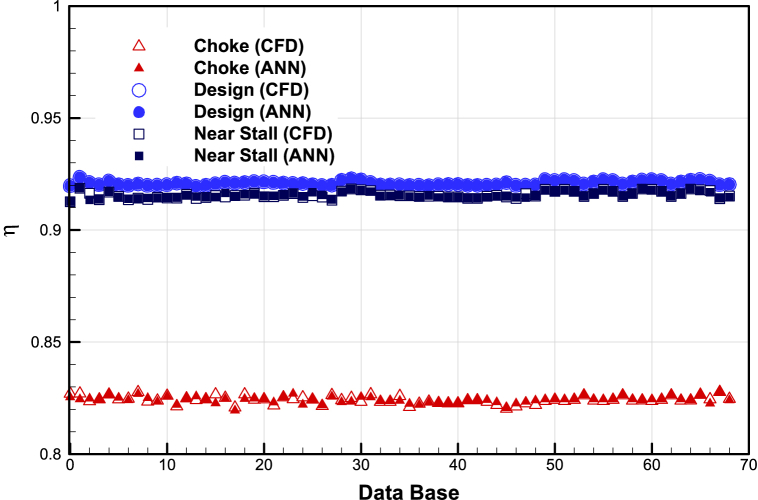
The prediction accuracy of adiabatic efficiency of ANN with CFD.

The prediction accuracy of artificial neural networks with CFD results is presented in Fig. 14, Fig. 15, and Fig. 16. The details of the ANN setting are given in Table 4.

**Table 4:** The Artificial Neural Networks specification.

Criteria	Function/Value
**Layers number**	3
**Neurons number**	24
**Feeding method**	Back propagation
**Epochs number**	500
**Convergence goal**	1e-16
**Output function**	Liner function
**Neuron activation function**	Sigmoid
**Data division**	Random
**Training algorithm**	Levenberg-Marquardt
**Performance**	Mean squared error

**Fig. 17:**
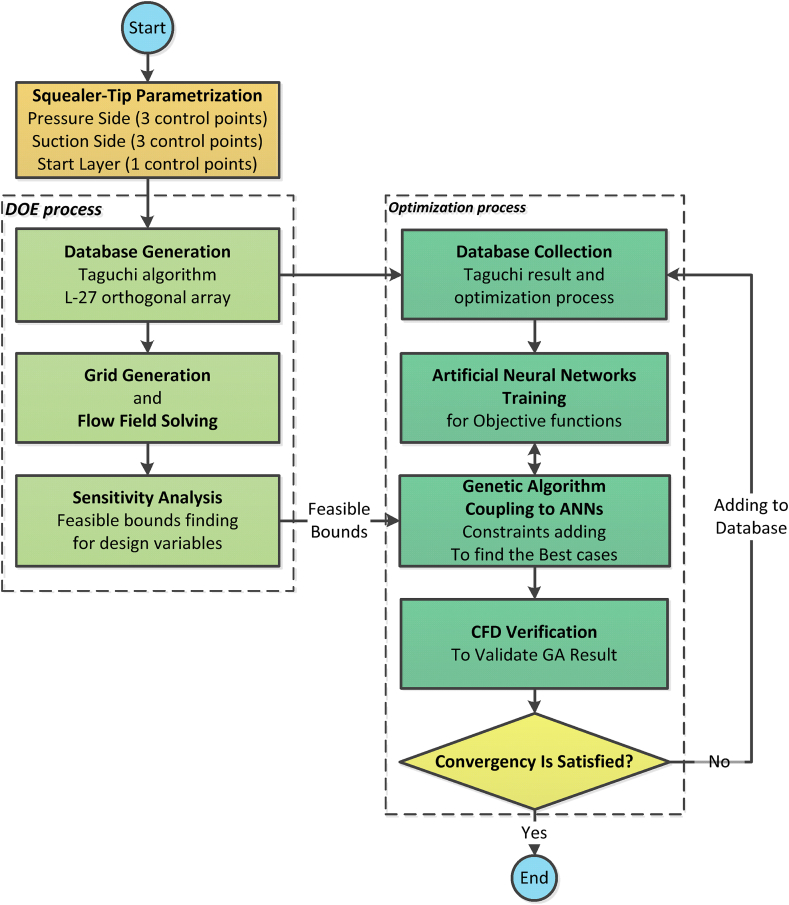
The flowchart of optimization.

In the optimization process of this research, the genetic algorithm is coupled with neural networks to search for optimal points, and the results are verified by CFD in the optimization loop. Therefore, if the error between results obtained from neural networks and CFD were higher than the optimal loop error range, the CFD results are sent back to the initial database for improving the neural network's capabilities. If the error between CFD and neural network results is less than the convergence error, the optimization loop ends, and the CFD results along with genetic algorithm selection coefficients for the optimal point are selected. The DOE process and the sensitivity analysis of design variables, as well as the numerical optimization process, are illustrated in Fig. 17 flowchart. Further details of the genetic algorithm are presented in Table 5.

**Table 5:** The Genetic Algorithm specification.

Criteria	Function/Value
**Population size**	100
**Generations number**	100
**Convergence criteria**	1e-6
**Mutation**	Uniform (0.1)
**Selection**	Uniform
**Crossover**	Two-point crossover
**Fitness scaling**	Rank

### 5.2 penalties and objective function

Using a penalty is a common method to strategize the results towards improvement, and prevent the reduction of the performance values compared to the reference values of Rotor-67. Therefore, the penalties of Equations (10), (11), (12) are set in the objective function of the genetic algorithm.

(10) $$Penalty_{{\dot{m}}_{Ch}}=10^{7}\times {\left(\max\;\left(0,\left({\dot{m}}_{Ch_{R67}}-{\dot{m}}_{Ch}\right)\right)\right)}^{2}\;\;Penalty_{{\dot{m}}_{D}}=10^{7}\times {\left(\max\;\left(0,\left({\dot{m}}_{D_{R67}}-{\dot{m}}_{D}\right)\right)\right)}^{2}\;\;Penalty_{{\dot{m}}_{NS}}=10^{7}\times {\left(\max\;\left(0,\left({\dot{m}}_{D_{R67}}-{\dot{m}}_{NS}\right)\right)\right)}^{2}$$

(11) $$Penalty_{{\mathrm{PR}}_{Ch}}=10^{7}\times {\left(\max\;\left(0,\left({\mathrm{PR}}_{Ch_{R67}}-{\mathrm{PR}}_{Ch}\right)\right)\right)}^{2}\;\;Penalty_{{\mathrm{PR}}_{D}}=10^{7}\times {\left(\max\;\left(0,\left({\mathrm{PR}}_{D_{R67}}-{\mathrm{PR}}_{D}\right)\right)\right)}^{2}\;Penalty_{{\mathrm{PR}}_{NS}}=10^{7}\times {\left(\max\;\left(0,\left({\mathrm{PR}}_{D_{R67}}-{\mathrm{PR}}_{NS}\right)\right)\right)}^{2}$$

(12) $$Penalty_{\eta_{Ch}}=10^{7}\times {\left(\max\;\left(0,\left(\eta_{Ch_{R67}}-\eta_{Ch}\right)\right)\right)}^{2}\;\;Objective_{\eta_{D}}=10\times \eta_{D}\;\;Objective_{\eta_{NS}}=10\times \eta_{NS}$$

Improving the stall efficiency and preventing reduction of the choke efficiency is one of the most important research goals, and therefore, a coefficient has been used in the equation of objective function in Equation (13).

(13) $${Objective=(Penalty}_{{\dot{m}}_{Ch}}+{Penalty}_{{\dot{m}}_{D}}+{Penalty}_{{\dot{m}}_{NS}})+\left({Penalty}_{{\mathrm{PR}}_{Ch}}+{Penalty}_{{\mathrm{PR}}_{D}}+{Penalty}_{{\mathrm{PR}}_{NS}}\right)+\left({Penalty}_{\eta_{Ch}}+{Objective}_{\eta_{D}}+{Objective}_{\eta_{NS}}\right)$$

## 6 result and discussion

**Fig. 18:**
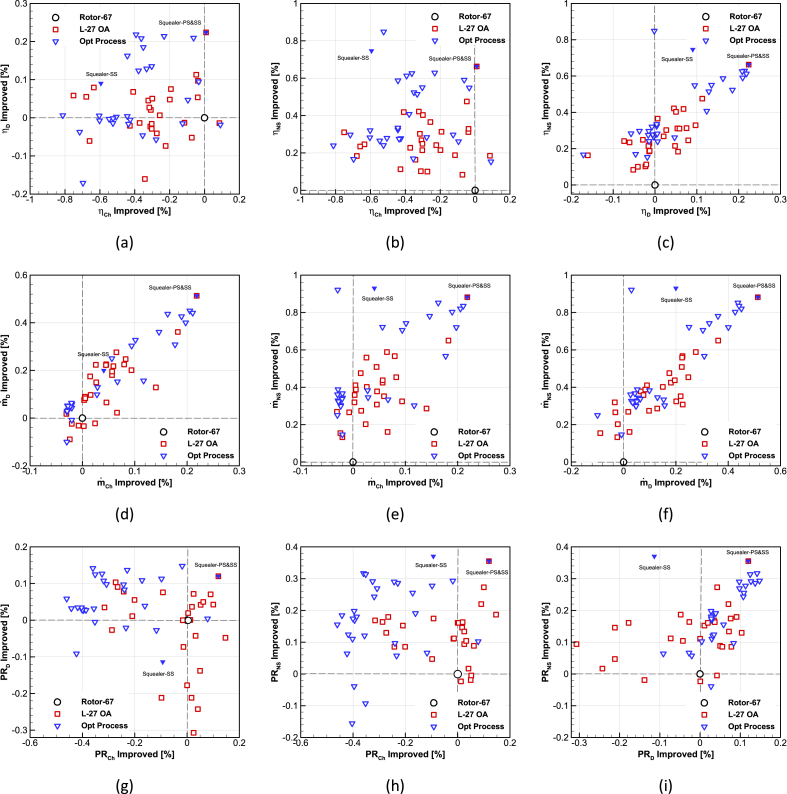
The percentage improvement of database values compared to NASA Rotor-67 for mass flow rate, total pressure ratio and adiabatic efficiency in three points of Choke, design and near the stall $\left(a\right)\;\eta_{Ch}-\eta_{D}\;\left(b\right)\;\eta_{Ch}-\eta_{NS}\;\left(c\right)\;\eta_{D}-\eta_{NS}\;\left(d\right)\;{\dot{m}}_{Ch}-{\dot{m}}_{D}\;\left(e\right)\;{\dot{m}}_{Ch}-{\dot{m}}_{NS}\;\left(f\right)\;{\dot{m}}_{D}-{\dot{m}}_{NS}\;\left(g\right)\;{\mathrm{PR}}_{Ch}-{\mathrm{PR}}_{D}\;\left(h\right)\;{\mathrm{PR}}_{Ch}-{\mathrm{PR}}_{NS}\;\left(i\right)\;{\mathrm{PR}}_{D}-{\mathrm{PR}}_{NS}$.

In the optimization process, a database of 27 arrays was used and then GA coupled with ANNs was solved. In this process, two categories of squealer tips were studied. The first category is suction-side squealer, and the second one is double-sided squealer. The results for the squealer-tip blades compared to original Rotor-67 blade are presented in Fig. 18. Finally, the two best optimal cases have been selected from this database and their characteristic maps have been extracted.

### 6.1 performance map

**Fig. 19:**
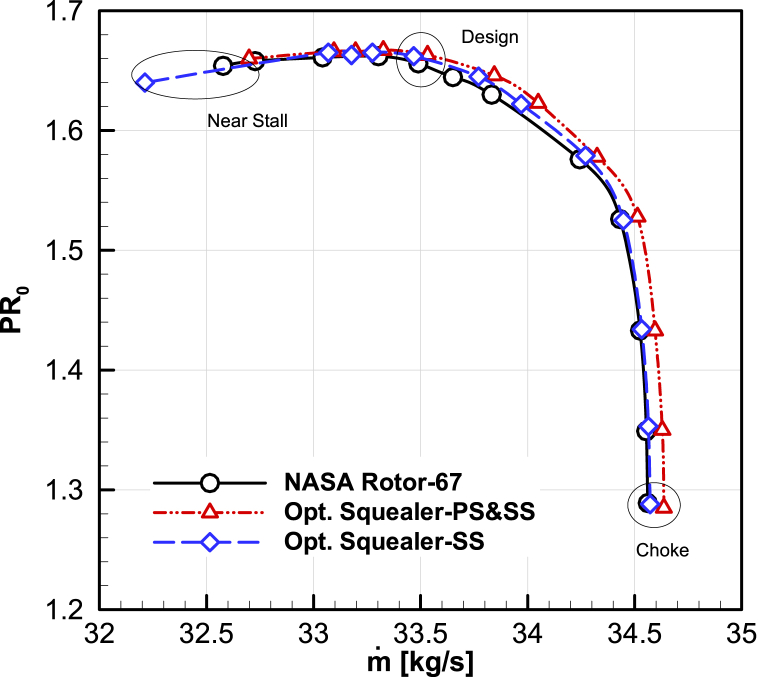
The comparison of optimized maps for total pressure ratio vs. mass flow rate.

**Fig. 20:**
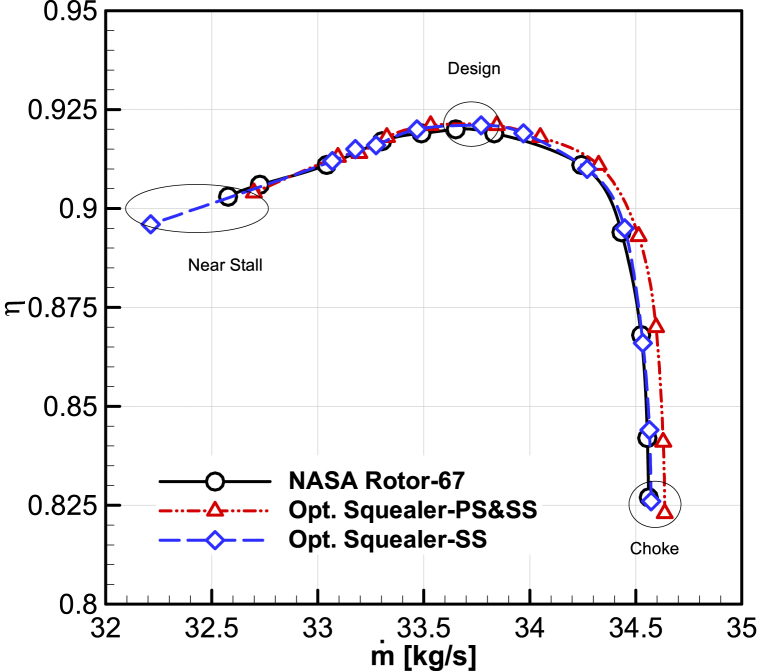
The comparison of optimized maps for adiabatic efficiency vs. mass flow rate.

The performance maps for the design speed of the Rotor-67 and two optimized compressors with squealer-tip blade have been calculated and presented in Fig. 19, Fig. 20. Additionally, the performance improvement of optimal squealer geometries for three in choke, design and near stall working areas are presented in Table 6, Table 7, Table 8. The increase in choke mass flow rate in double-sided squealer and surge margin improvement using suction-side squealer is obvious from the figures.

**Table 6:** Optimization result of performance values for choke point.

Choked point	Cases	NASA Rotor-67	Optimized Squealer-PS&SS	Optimized Squealer-SS
**Performance values**	**Mass flow rate [kg/s]**	34.561	34.636	34.576
	**Total pressure ratio**	1.289	1.285	1.285
	**Adiabatic efficiency**	0.827	0.823	0.821
**Improvement**	**Mass flow rate [%]**	–	0.216	0.042
	**Total pressure ratio [%]**	–	−0.289	−0.285
	**Adiabatic efficiency [%]**	–	−0.433	−0.722

**Table 7:** Optimization result of performance values for design point.

Design point	Cases	NASA Rotor-67	Optimized Squealer-PS&SS	Optimized Squealer-SS
**Performance values**	**Mass flow rate [kg/s]**	33.651	33.844	33.787
	**Total pressure ratio**	1.640	1.646	1.645
	**Adiabatic efficiency**	0.920	0.921	0.920
**Improvement**	**Mass flow rate [%]**	–	0.575	0.405
	**Total pressure ratio [%]**	–	0.361	0.327
	**Adiabatic efficiency [%]**	–	0.189	0.057

**Table 8:** Optimization result of performance values for near stall point.

Near Stall point	Cases	NASA Rotor-67	Optimized Squealer-PS&SS	Optimized Squealer-SS
**Performance values**	**Mass flow rate [kg/s]**	32.577	32.699	32.594
	**Total pressure ratio**	1.654	1.660	1.648
	**Adiabatic efficiency**	0.903	0.904	1.170
**Improvement**	**Mass flow rate [%]**	–	0.374	0.053
	**Total pressure ratio [%]**	–	0.363	−0.362
	**Adiabatic efficiency [%]**	–	0.111	0.177

According to the results, the improvement of the total pressure ratio, adiabatic efficiency and corrected mass flow rate have been obtained in the design and near stall areas when squealer is implemented on both sides, with more significant effects in near stall regions. When side squealer is used, besides acquiring increased mass flow rate and efficiency, total pressure ratio is considerably reduced.

### 6.2 surge margin

Surge margin is one of the important stability parameters of a compressor, showing a safe operating distance between operating point and surge point, which is calculated according to Equation (14). Table 9 presents these results which show that the surge margin is improved in the optimal squealer blade.

(14) $$SM\;\left[\%\right]=\left(1-\left(\frac{{\mathrm{PR}}_{D}}{{\mathrm{PR}}_{NS}}\right)\times \left(\frac{{\dot{m}}_{NS}}{{\dot{m}}_{D}}\right)\right)\times 100$$

**Table 9:** The values and improvement of surge margin of optimal rotors in comparison with Rotor-67.

Cases	NASA Rotor-67	Optimized Squealer - PS & SS	Optimized Squealer – SS
**Surge margin**	4.017	4.210	4.279
**Improvement [%]**	–	4.806	6.538

### 6.3 optimal rotor tip

**Fig. 21:**
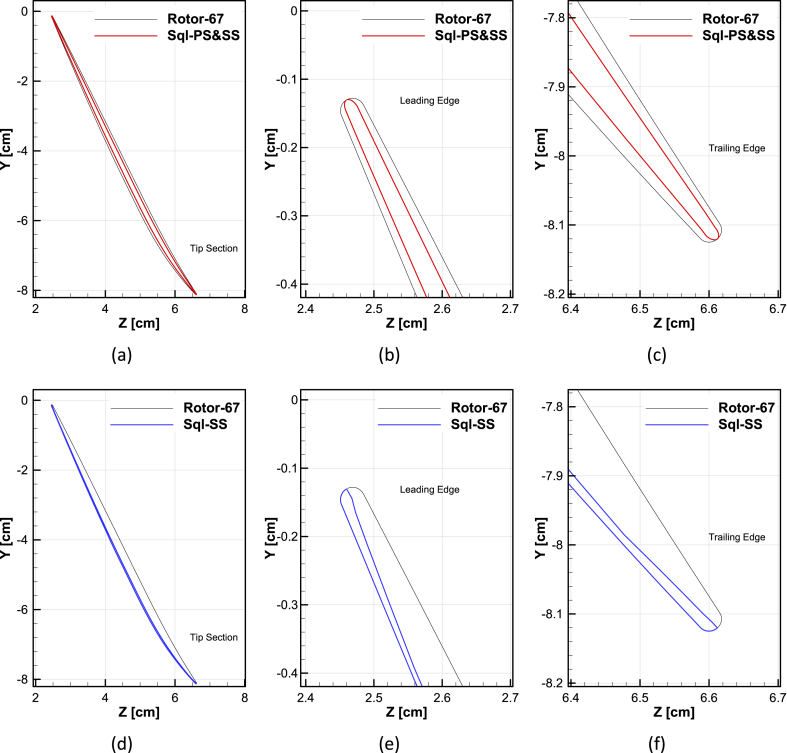
Schematic views of the squealer at a tip section, (a) Squealer-PS&SS, (b) LE of Squealer-PS&SS, (c) TE of Squealer-PS&SS, (d) Squealer-SS, (e) LE of Squealer-SS, (f) TE of Squealer-SS.

A sample of squealer tip geometry in the blade-to-blade view is demonstrated in Fig. 21. The figures demonstrated a detailed view of the optimized squealer geometries for the double-sided and suction-side squealer. Furthermore, the obtained values for optimal geometries are presented in Table 10. Ellipse is used to form the blade tip leading and trailing edge, in the suction side case, to further refine and adjust the incoming flow.

**Table 10:** Minimum and maximum values of blade squealer-tip coefficients.

	Coefficients	NASA Rotor-67	Optimized Squealer-PS&SS	Optimized Squealer-SS
**Pressure side**	a_1_	1.00	0.70	0.38
	a_2_	1.00	0.60	0.40
	a_3_	1.00	0.50	0.51
**Suction side**	a_4_	1.00	0.70	1.0
	a_5_	1.00	0.60	1.0
	a_6_	1.00	0.50	1.0
**Span**	a_7_	1.00	0.94	0.97

### 6.4 radial profiles

**Fig. 22:**
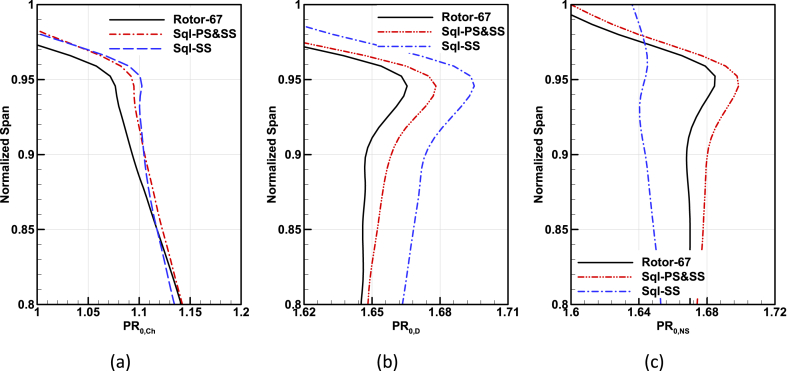
Comparison of the total pressure ratio in spanwise direction for Rotor-67 and optimal geometries, (a) Choke operating point, (b) Design point, (c) Near-Stall operating point.

**Fig. 23:**
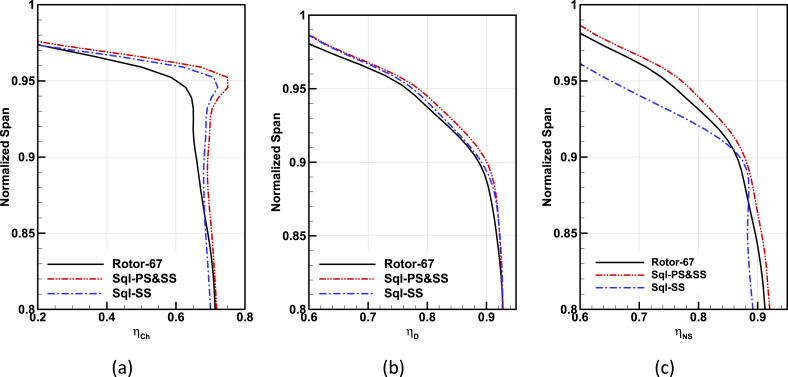
Comparison of the adiabatic efficiency in spanwise direction for Rotor-67 and optimal geometries, (a) Choke operating point, (b) Design point, (c) Near-Stall operating point.

The distribution of total pressure ratio and adiabatic efficiency in the radial direction just after the compressor rotor for Rotor-67 base geometry and two obtained optimal geometries are extracted in three different operating conditions and are depicted in Fig. 22, and Fig. 23.

According to the results, the effects of the squealer are clear in the improvement of the three performance parameters of on-design Total pressure ratio and adiabatic efficiency of blade tip areas (span 0.9 to 1); while it has less decreasing of adiabatic efficiency at near stall region (for squealer-SS).

### 6.5 blade loading

**Fig. 24:**
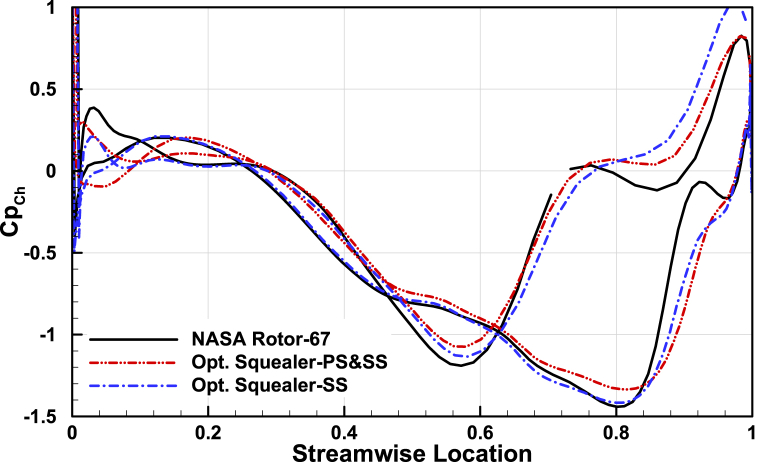
The comparison of pressure coefficient at tip section for Rotor-67 and optimal rotors for choked point.

**Fig. 25:**
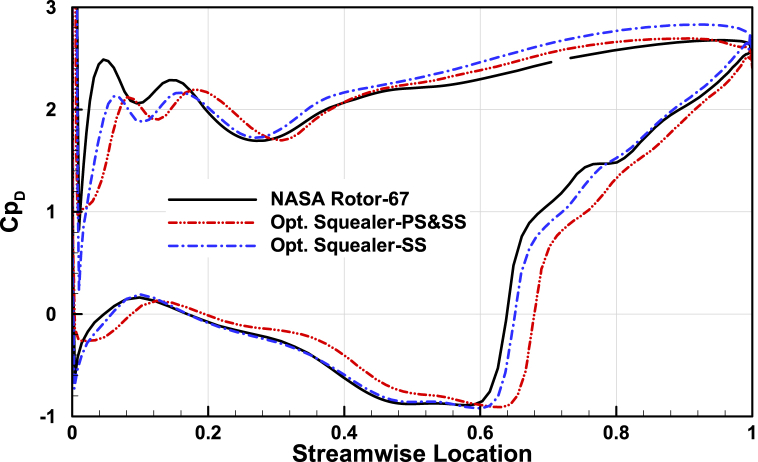
The comparison of pressure coefficient at tip section for Rotor-67 and optimal rotors for design pint.

**Fig. 26:**
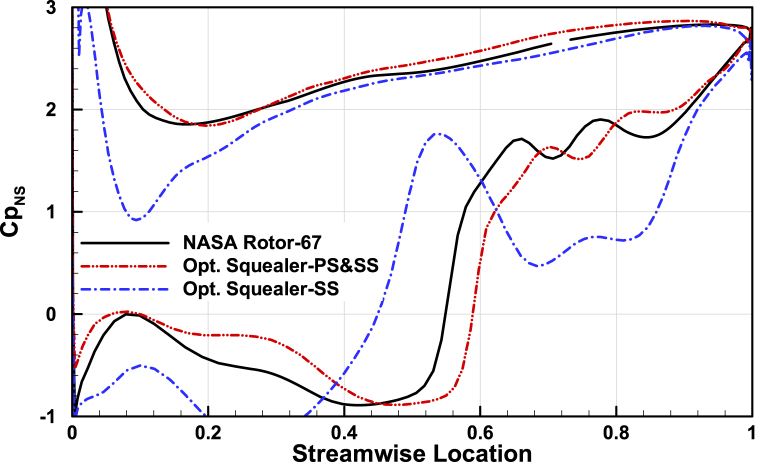
The comparison of pressure coefficient at tip section for Rotor-67 and optimal rotors for near stall point.

Change of geometry results in different pressure distribution over the blade tip area, thus to better compare the parameter in optimized squealers and the baseline geometry, pressure coefficient distribution in blade tip section is presented in Fig. 24, Fig. 25, Fig. 26 for three operating points. The double-side squealer geometry acts more like the baseline geometry and minor changes are present. But for the suction side squealer, since a considerable amount of thickness is lost the changes in pressure distribution are significant, mainly in near stall operating point. This has finally resulted in better surge margin based on the results.

### 6.6 tip leakage

The results of the aerodynamic values of the optimized squealer and the Rotor-67 in the blade tip region are presented put into comparison in this section.

#### 6.6.1 tip-leakage map

**Fig. 27:**
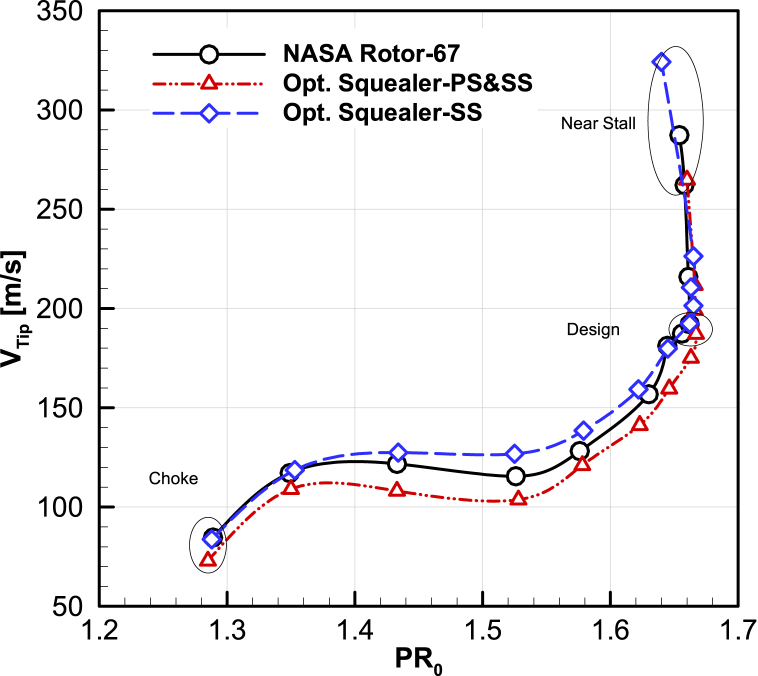
The comparison of optimized maps for total pressure ratio vs. average tip leakage velocity.

**Fig. 28:**
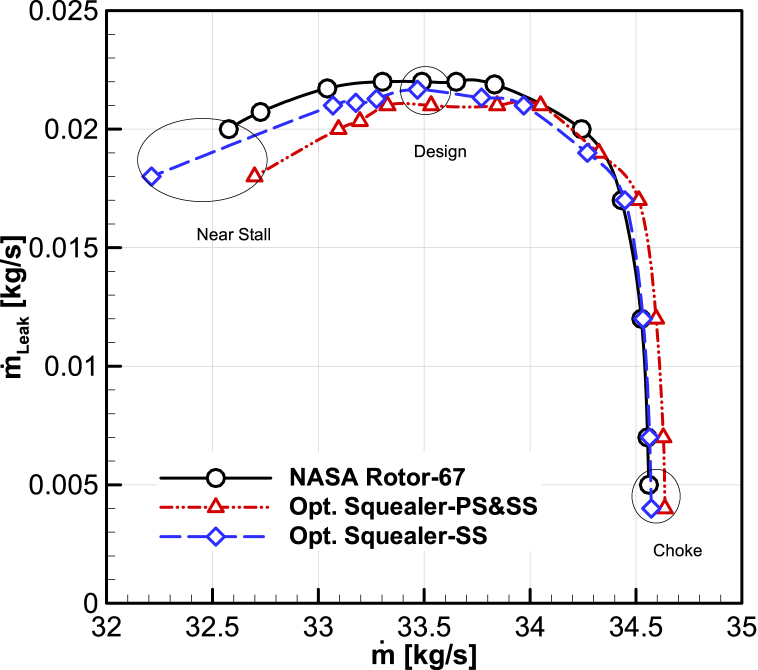
The comparison of optimized maps for mass flow rate vs. tip mass flow rate.

The tip leakage mass flow rate-inlet mass flow rate and tip velocity-Total pressure ratio curves have been extracted for the design speed, and demonstrated in Fig. 27, Fig. 28. According to the results of two optimal rotors, the leakage mass flow rate in design and near the stall points are lower than the leakage mass flow rate of the Rotor-67.

#### 6.6.2 tip-leakage contour

**Fig. 29:**
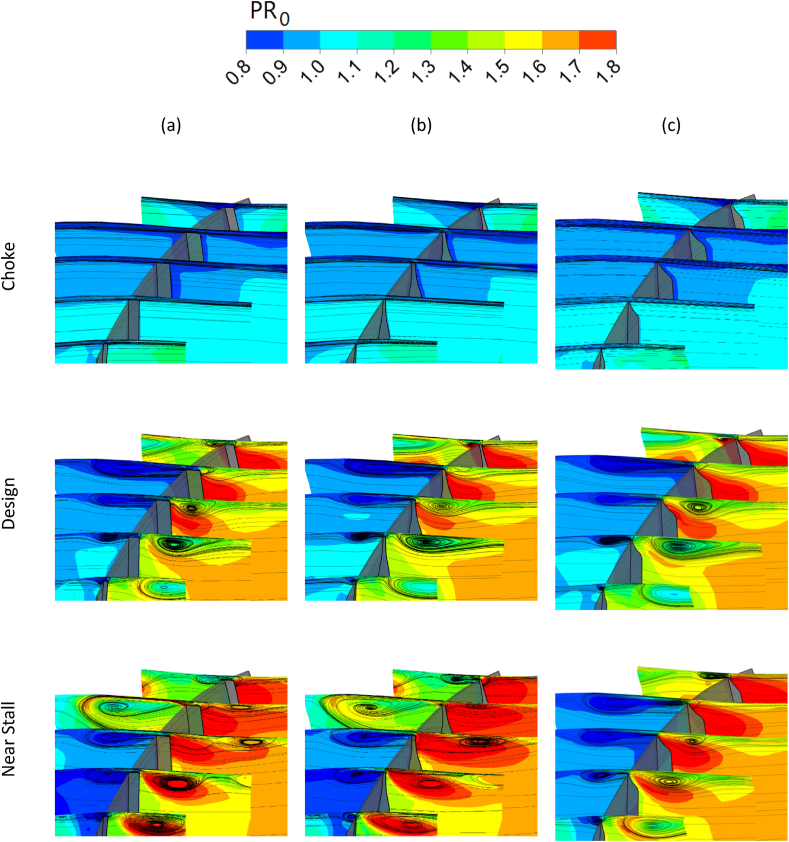
The comparison of Tip-leakage streamlines of optimized squealer with Rotor-67, Column (a) NASA Rotor-67, Column (b) Optimized Squealer-PS&SS, Column (c) Optimized Squealer-SS.

The blade tip vortices are presented using the streamlines of the blade tip leakage in five planes perpendicular to the suction and pressure surfaces. Fig. 29 demonstrates these streamlines for the optimal squealer rotors compared to the Rotor-67, in three operating regions.

**Fig. 30:**
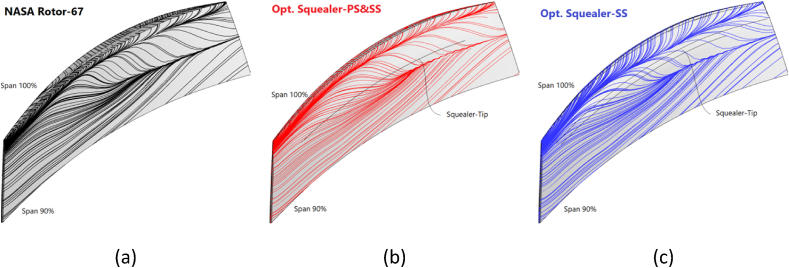
Schematic view of surface streamlines in blade tip region (90 %–100 % blade span) for Rotor-67 and optimal geometries, (a) NASA Rotor-67, (b) Optimized Squealer-PS&SS, (c) Optimized Squealer-SS.

The tip vortex generated at the design and near stall operating points has a greater depth and a smaller width than the vortex in optimal blades which are subsequently created because of the presence of squealer-tip. Therefore, these vortices are the important reason for the reduction of secondary flows and improvement of performance parameters in spanwise direction. The reduction of vortex propagation in the suction side region in blades with squealer tips results in less deviation of flow from the sold surface of the rotor and less dissipation of energy. This has been addressed in velocity curl plots in the next section. Additionally, Fig. 30 demonstrates 3-dimensional surface streamlines in tip region of the near stall operating point.

**Fig. 31:**
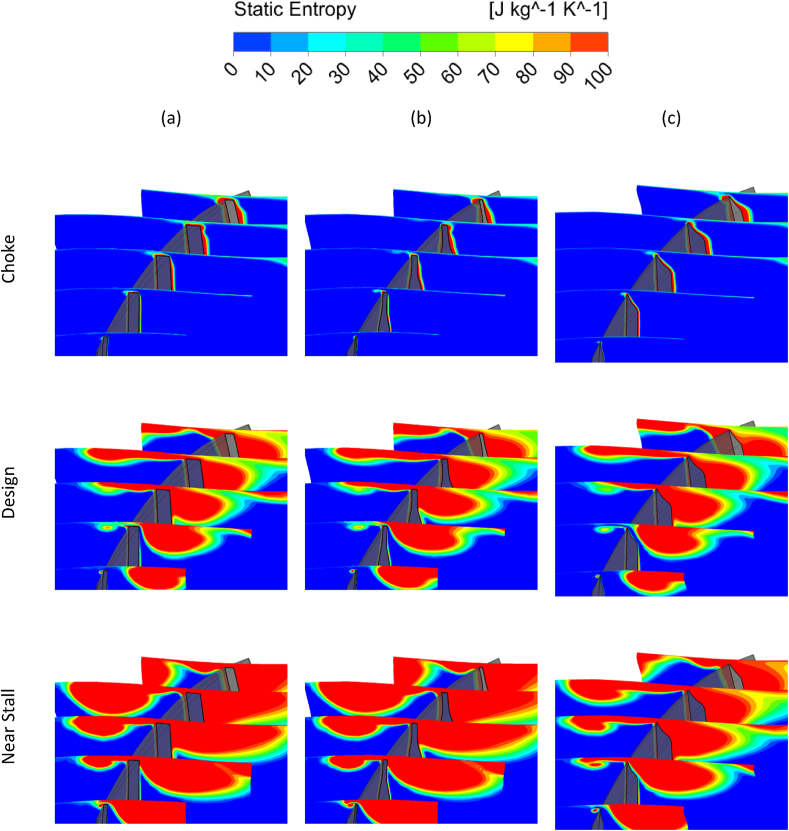
The comparison of Tip-Entropy of optimized squealer with Rotor-67, Column (a) NASA Rotor-67, Column (b) Optimized Squealer-PS&SS, Column (c) Optimized Squealer-SS.

By comparing the entropy contour of the blade tip in Fig. 31, it also seems that the decrease in relative Mach has caused a decrease in the entropy production in the optimal squealer geometries compared to the Rotor-67. The figures depict that the entropy increase due to presence of squealer is reduced because of the changed mixing loss, affecting the flow structure in suction side. The main region of entropy increase is downstream of the 60 % streamwise location. This positive effect of squealer is more evident in near stall working points, where high Total pressure ratio drives more flow through the tip clearance. The suction side squealer shows a more promising result in reducing the mixing loss.

### 6.7 circumferential distribution

**Fig. 32:**
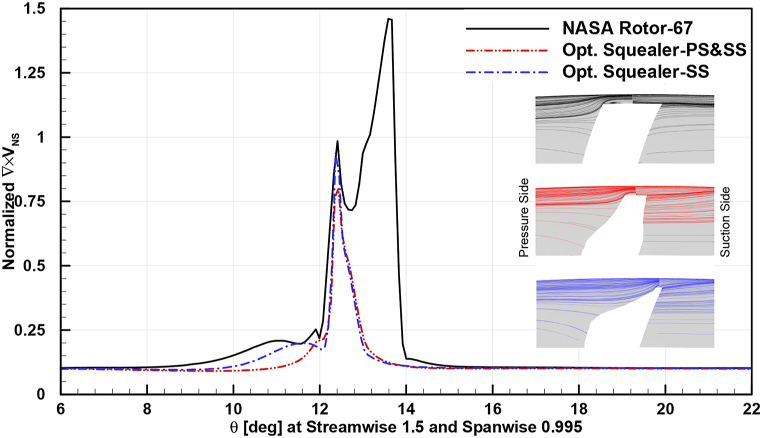
Comparison of normalized choke velocity curl for Rotor-67 and optimal rotors.

**Fig. 33:**
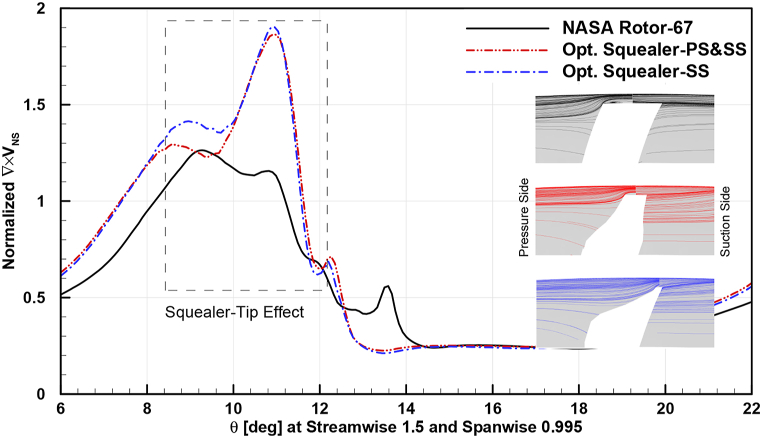
Comparison of normalized design velocity curl for Rotor-67 and optimal rotors.

**Fig. 34:**
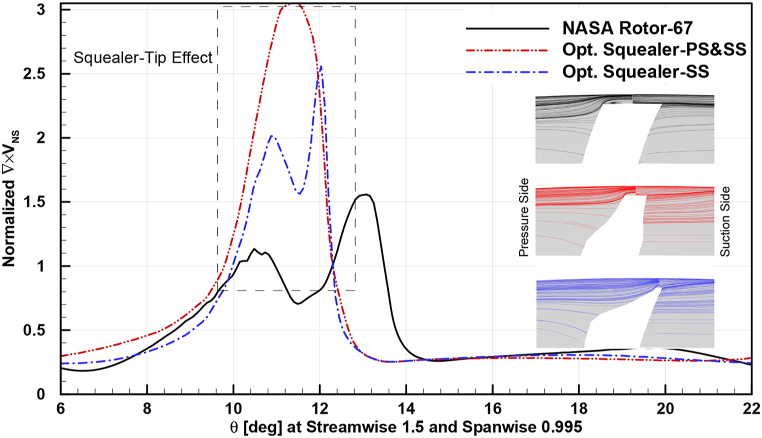
Comparison of normalized near stall velocity curl for Rotor-67 and optimal rotors.

In Fig. 32, Fig. 33, Fig. 34, circumferential distribution of normalized velocity curl vectors at 0.95 spanwise and 1.5 streamwise locations are plotted. The parameter distribution is demonstrated in design and near stall operating points for baseline and obtained optimum squealer geometries.

As it is depicted, the velocity curl is increased in presence of SS and PS-SS squealers, compared to baseline geometry, which is due to creation of additional vortices. This causes a sealing like effect in blade tip area by increasing energy dissipation of the leakage flow and consequently tip leakage flow is reduced. The aforementioned increase in surge margin is another effect caused by this sealing effect. The higher velocity curl is a result of additional vortex effects in the tip area and as mentioned before, by changing the flow structure and mixing loss, the tip leakage is reduced and the suction side flow can follow the solid geometry in a better manner and a more desired flow exit angle at the trailing edge.

### 6.8 streamwise distribution

**Fig. 35:**
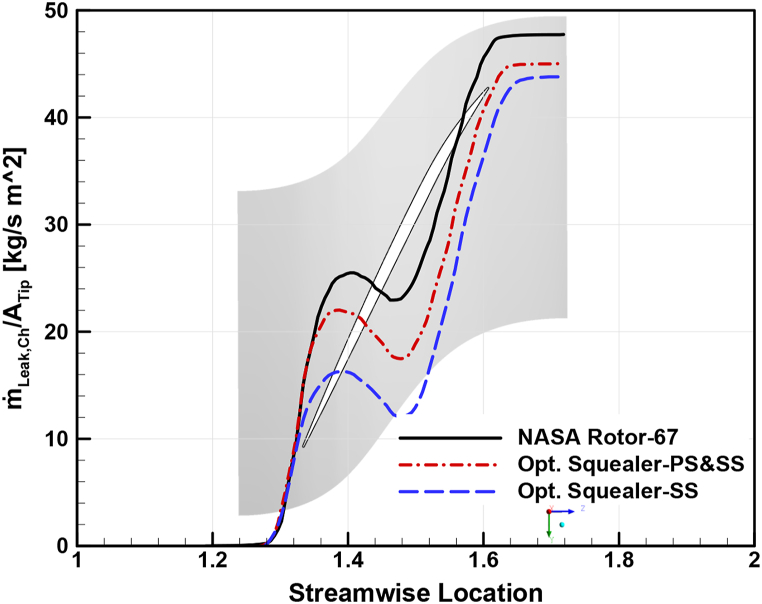
Leakage mass flow rate per tip clearance area at choke point for Rotor-67 and optimal rotors.

**Fig. 36:**
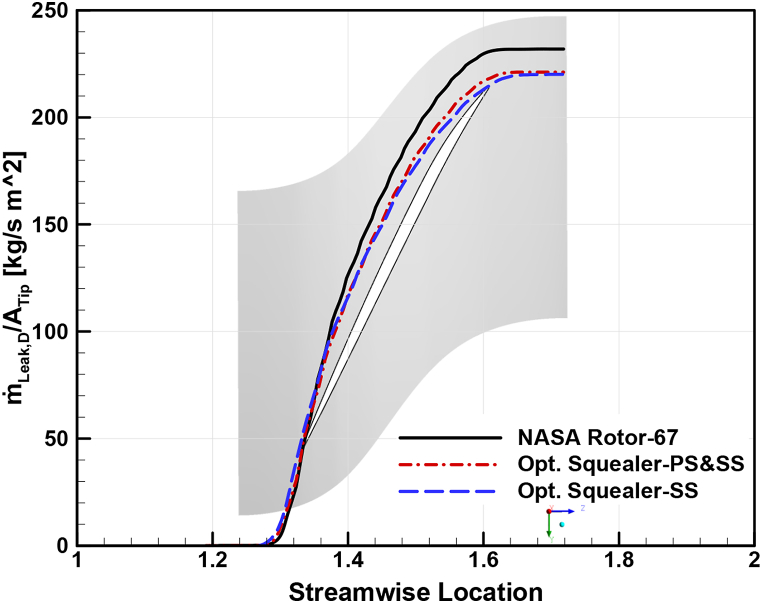
Leakage mass flow rate per tip clearance area at design point for Rotor-67 and optimal rotors.

**Fig. 37:**
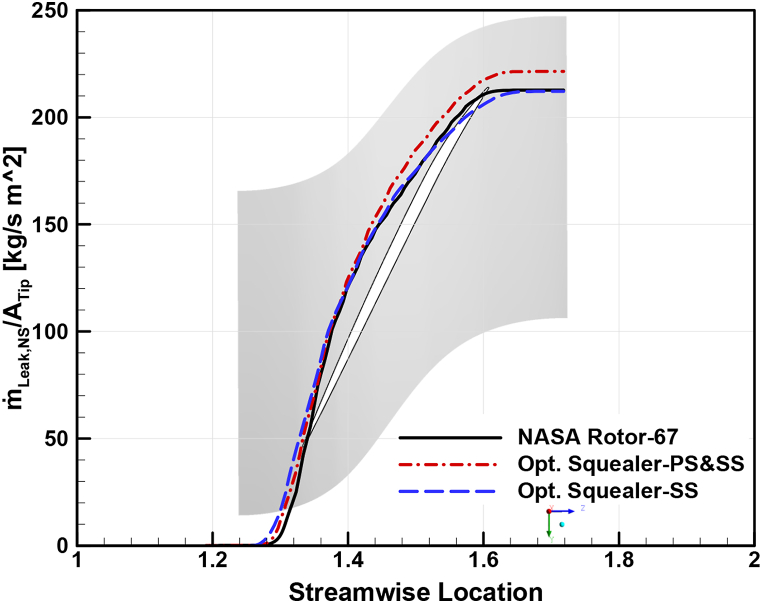
Leakage mass flow rate per tip clearance area at near stall point for Rotor-67 and optimal rotors.

To analyze the effects of squealer on the secondary airflow in the tip region of the optimized rotors compared to Rotor-67 more thoroughly, leakage mass flow rate distribution in streamwise direction, through a fixed cross-sectional surface from the LE to is used. These results have been carried out for choke, design, and near stall conditions, as shown in Fig. 35, Fig. 36, Fig. 37, respectively. As expected, the leakage mass flow in both optimized geometries reduced compared to the reference geometry, resulting in reduced losses, improved efficiency, and increased surge margins.

## 7 conclusion

The study explores the use of squealer tip to improve the performance of axial compressor blades in various operating conditions in a specified tip clearance similar to baseline geometry. Splines with control points are utilized to modify the geometry of the squealer tip, both on the suction and pressure surfaces. A design of experiments (DoE) approach is used to analyze the impact of these modifications on aerodynamic performance. Neural networks are trained using the data generated in the DoE, and they are integrated with an optimization algorithm to identify optimal performance areas. The research shows that these modifications reduce blade tip leakage flow and weaken tip vortices, leading to improved performance parameters in compressor rotor operation. The main results of the study are summarized below.

• For the choke operating region, the optimum double-sided and suction side squealers resulted in an increase in mass flow rate by 0.216 and 0.042%, respectively, but total pressure ratio and adiabatic efficiency were negatively affected.
• In the design point, all main performance parameters were considerably improved; 0.575 and 0.405% increase for mass flow rate of double-sided and suction side squealer, for example. In this matter, data for other parameters and working points are presented in Table 6 to Table 8.
• A promising effect of squealer tip is found to be its effect on surge margin improvement, in this research is 4.8 % for double-sided and 6.5 % for suction side squealer.
• Entropy increase is reduced in the presence of a squealer tip, mainly after a 60 % streamwise location where a significant entropy increase exists.
• Tip leakage flow is significantly reduced when an optimum shape of squealer is employed, mainly in the choke and design point, resulting in an increase overall mass flow rate of the compressor.

## Funding

There is no funding for this research.

## Data availability statement

The data that support the findings of this study are available on request from the corresponding author.

## CRediT authorship contribution statement

**Mojtaba Heidarian Shahri:** Writing - original draft, Visualization, Validation, Software, Resources, Methodology, Investigation, Formal analysis, Data curation. **Saeid Habibzadeh:** Writing - original draft, Visualization, Validation, Software, Resources, Methodology, Investigation, Formal analysis, Data curation. **Ali Madadi:** Writing - review & editing, Supervision, Resources, Project administration, Formal analysis, Data curation, Conceptualization.

## Declaration of competing interest

The authors declare that they have no known competing financial interests or personal relationships that could have appeared to influence the work reported in this paper.
